# Notice

**Published:** 1885-04

**Authors:** 


### "NOTICE."

We desire to call special attention to the modest advertisement of a dentist who desires to change his location, and which will be found occupying a modest place in the advertising pages. We know the advertiser, and can assure any one who has a place such as he desires, that a rare opportunity is offered for securing a coadjutor who is all that a first-class dentist could desire. Although a young man, he has considerable experience, with ability, intelligence and skill of a high order, and he is far from being unknown in the dental profession.

